# Association between Hypertension and Periodontitis: Possible Mechanisms

**DOI:** 10.1155/2014/768237

**Published:** 2014-01-08

**Authors:** Xin-Fang Leong, Chun-Yi Ng, Baharin Badiah, Srijit Das

**Affiliations:** ^1^Department of Pharmacology, Faculty of Medicine, Universiti Kebangsaan Malaysia, Jalan Raja Muda Abdul Aziz, 50300 Kuala Lumpur, Malaysia; ^2^Department of Clinical Oral Biology, Faculty of Dentistry, Universiti Kebangsaan Malaysia, Jalan Raja Muda Abdul Aziz, 50300 Kuala Lumpur, Malaysia; ^3^Department of Periodontology, Faculty of Dentistry, Universiti Kebangsaan Malaysia, Jalan Raja Muda Abdul Aziz, 50300 Kuala Lumpur, Malaysia; ^4^Department of Anatomy, Faculty of Medicine, Universiti Kebangsaan Malaysia, Jalan Raja Muda Abdul Aziz, 50300 Kuala Lumpur, Malaysia

## Abstract

This review is to examine the current literatures on the relationship between periodontitis and hypertension as well as to explore the possible biological pathways underlying the linkage between these health conditions. Hypertension is one of the major risk factors for cardiovascular diseases. Oxidative stress and endothelial dysfunction are among the critical components in the development of hypertension. Inflammation has received much attention recently and may contribute to a pivotal role in hypertension. Periodontitis, a chronic low-grade inflammation of gingival tissue, has been linked to endothelial dysfunction, with blood pressure elevation and increased mortality risk in hypertensive patients. Inflammatory biomarkers are increased in hypertensive patients with periodontitis. Over the years, various researches have been performed to evaluate the involvement of periodontitis in the initiation and progression of hypertension. Many cross-sectional studies documented an association between hypertension and periodontitis. However, more well-designed prospective population trials need to be carried out to ascertain the role of periodontitis in hypertension.

## 1. Introduction

The periodontal diseases are a group of chronic inflammatory diseases, involving the soft tissue and bone surrounding the teeth in the jaws, or known as periodontium. Periodontal diseases including gingivitis and periodontitis are among the most common dental diseases after tooth decay in humans. Periodontal diseases are characterized by inflammation of tooth-supporting tissues caused by bacterial infection [1]. Gingivitis is a very common reversible condition, which manifests as redness, gum swelling, and bleeding during tooth-brushing and flossing. Gingivitis may progress into periodontitis with further destruction of periodontal tissues ligament and alveolar bone if left without appropriate treatment. Teeth may become mobile and eventually be exfoliated following the diminution of periodontal supporting tissues [2]. This process is attributed to the release of toxic products from the pathogenic bacteria plaque in addition to the inflammation of gingival tissues elicited by the host response [3–6].

Periodontitis is linked to an increased risk of cardiovascular diseases (CVD). The chronic inflammatory process of periodontitis and the host response provide the basis for the hypothetical association between periodontitis and CVD [7, 8]. Hypertension increases the risk of various adverse cardiovascular events such as atherosclerosis, stroke, and coronary heart disease. Oxidative stress and endothelial dysfunction have been hypothesized to be involved in the pathogenesis of hypertension. It is well known that hypertension and periodontitis share common risk factors, namely, smoking, stress, increased age, and socioeconomic factors. These risk factors may confound the association between hypertension and periodontitis. Nevertheless, according to the scientific statement issued by the American Heart Association (AHA) published in *Circulation*, observational studies support an association between periodontal disease and cardiovascular disease, independent of shared risk factors [9].

Although current epidemiological data are yet to provide sufficient evidence to prove a causal relationship between these two diseases, researchers have identified chronic inflammation as an independent link of periodontal disease in the development and progression of CVD in some patients [10]. Both AHA and the American Academy of Periodontology (AAP) were in agreement that more thorough and long-term interventional studies should be carried out in order to gain an in-depth knowledge of the diseases' mechanism. The present review is to examine the existing literature on the association between hypertension and periodontitis. In addition, we looked into the possible mechanisms to explain this link.

## 2. Role of Inflammation in Hypertension 

Inflammation is an essential component of immune response to pathogens, damaged cells, and other potent inflammatory stimuli including reactive oxygen radicals. While it provides a pivotal defense mechanism against injurious agents, inflammation itself may cause injury to surrounding healthy bystander cells at the site. Inflammation is therefore a ‘double-edged sword' as this adaptive response might eventually become maladaptive after a chronic time. In blood vessel, inflammation increases vascular permeability and alters cytoskeletal elements in endothelial cells, disrupting the endothelial functions in controlling vascular health. Hence, there is a potential association between vascular inflammation and hypertension.

Over the past three decades, the role of vascular inflammation as a mechanism that participates in the progression of hypertension has gained increasingly strong footing through a tremendous amount of supportive reports [11–13]. Khraibi et al. [14] have found that chronic immunosuppressive therapy with cyclophosphamide significantly attenuated blood pressure (BP) elevation in Okamoto spontaneously hypertensive rats (SHR). This finding supported the hypothesis regarding the involvement of inflammatory reaction in hypertension. In the following year, Norman et al. [15] demonstrated that the development of hypertension was delayed by correcting the immune imbalance state in SHR. Their works continued to show that immunological dysfunction is one of the key aetiologies of hypertension [16]. Dzielak [17] pointed out an inflammatory involvement in hypertension by observing an alteration in the serum immunoglobulin levels in both patients and laboratory animals. Furthermore, the interaction between inflammatory cells and endothelial cells was increased in hypertensive patients [18]. Kampus et al. [19] also found an increase in C-reactive proteins (CRP) and vascular wall stiffness in untreated hypertensive patients. More recently, a link between hypertension and inflammatory responses to oxidized low-density lipoprotein was reported in patients, further suggesting that BP is directly correlated to immunological milieu [20].

The overall contribution of inflammation to vascular damage in hypertensive patients remains an interesting puzzle to be solved by scientists. Research during the last dozen years has shed light on some aspects of this puzzle. Endothelial cells, which line the intimal surface of blood vessel, are the primary target of immunological attack in inflammatory responses. Under normal conditions, the endothelium maintains a vasodilator, antithrombotic, and antiinflammatory state. However, a proinflammatory condition could contribute to endothelial dysfunction.

Certain inflammatory adhesion molecules are involved in the pathogenesis of hypertension and predictive of future cardiovascular events. Vascular cell adhesion molecule-1 (VCAM-1) and intercellular adhesion molecule-1 (ICAM-1) are expressed by the endothelial cells. Their expressions are upregulated in response to inflammatory insult [21]. The increased expression of adhesion molecules on endothelial cells is a common process in response to inflammation [22]. VCAM-1 and ICAM-1 are recognized as important cardiovascular risk markers [23, 24]. VCAM-1 and ICAM-1 mediate leukocytes binding to the endothelial lining.

**Figure 1:**
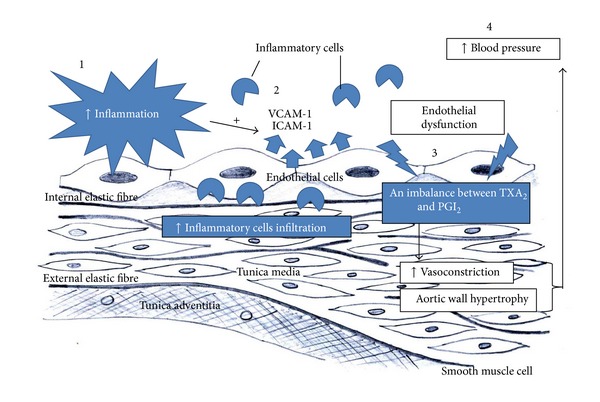
(1) Inflammatory stimuli induce the expression of cellular adhesion molecules such as vascular cell adhesion molecule-1 (VCAM-1) and intracellular adhesion molecule-1 (ICAM-1) on endothelial cells. (2) Increased VCAM-1 and ICAM-1 expressions recruit more leukocytes to the site of inflammation. (3) Leukocytes infiltration and production of cytokines lead to oxidative stress and inflammation, which result in endothelial dysfunction, affecting the balance of synthesis between vasodilators and vasoconstrictors. The imbalance favours vasoconstriction and adverse vascular remodelling, (4) consequently leading to elevation of blood pressure.

Increased leukocytes infiltration and production of cytokines exaggerate oxidative stress and inflammation, eventually causing a disturbance to the normal endothelial function in regulating BP. Endothelial cells play a crucial part in BP homeostasis through the synthesis of vasodilators such as nitric oxide (NO), prostacyclin (PGI_2_), and endothelium-derived hyperpolarising factor (EDHF) and vasoconstrictors such as endothelin-1, thromboxane (TXA_2_), and angiotensin II. During endothelial dysfunction, the balance between these vasodilators and vasoconstrictors is disturbed in favor of the latter. Ng et al. [25] have recently shown that the levels of VCAM-1 is directly associated with the imbalance between PGI_2_ and TXA_2_ in hypertensive rats. Therefore, inflammation may be involved in hypertension by directly damaging the endothelial BP regulation.  Figure 1 depicts the mechanism of elevating BP in linkage to vascular inflammation.

## 3. Evidences of Association between Hypertension and Periodontitis 

Hypertension is a major global health disorder affecting about 972 million adult populations in year 2000. This number is expected to grow to 1.56 billion by the year 2025 [26]. Prevalence of hypertension in most developing countries is comparable to the developed countries [27, 28]. Hypertension is defined when a patient has an elevated systolic BP greater than 140 mmHg and/or diastolic BP greater than 90 mmHg [29]. A patient with systolic BP ranging between 120 mmHg and 139 mmHg, and/or diastolic BP of 80 mmHg to 89 mmHg, is categorized as prehypertensive. Patients at this stage have the tendency to develop hypertension; hence medical approaches and life style need to be taken care of [29].

More than 700 species of bacteria are estimated to be found in the oral cavity [30, 31]. These bacteria form a symbiosis relationship while living at various oral sites [30, 31]. Periodontitis is often associated with extensive formation of dental plaques or better known as biofilms, at the tooth and the gingival interface [32]. The biofilms protect microbes from the host defense system and antimicrobial agents such as antibiotics. The biofilms are mainly dominated by anaerobic, Gram-negative bacteria, for example, *Porphyromonas gingivalis *(*P. gingivalis*), *Aggregatibacter actinomycetemcomitans,* and spirochetes like *Treponema denticola* [33–35].

Although bacterial biofilms are necessary for the development of periodontal disease, they are not the sole contributor to the disease. Hence, a susceptible host is required. Dental biofilms release a variety of biologically active products, including bacterial lipopolysaccharides, chemotactic peptides, protein toxins, and organic acids [36]. Production and release of proinflammatory prostaglandins and cytokines such as interleukin-1 beta (IL-1*β*), interleukin-6 (IL-6), interleukin-8 (IL-8), and tumour necrosis factor-alpha (TNF-*α*) are triggered in response to stimuli of dental biofilms [37, 38]. The active products by biofilms and host responses are responsible for periodontal tissue destruction. The released products may also affect various disease pathways such as atherosclerosis and mucosal inflammation [36].

Over the past years, studies carried out in patients with periodontal disease have related hypertension with chronic periodontal disease localized in the gingival tissues [39]. These studies documented that hypertensive subjects exhibited a more detrimental periodontal status [40–43]. Epidemiological studies to date have shown an association between hypertension and periodontitis (Table 1). Nevertheless, the related studies were mostly cross-sectional, with varied numbers of subject and assessment methods. For instance, earlier investigations depended on surrogate markers of exposure, including depth of periodontal pocket, attachment loss, and dental indices, or based on the number of missing tooth or self-reported periodontal status such as oral hygiene practice. As a result, data obtained from early studies need to be interpreted with caution, emphasizing the need for further research, as suggested by Tsioufis et al. [44].

## 4. Possible Linking Pathways for the Association between Hypertension and Periodontitis 

### 4.1. Inflammation

Hypertension and periodontitis are two diseases which seem to be profoundly unrelated. However, since periodontitis is a chronic infection that leads to inflammation, the appreciation of periodontitis as a risk factor for hypertension has burgeoned lately. There is now evidence that supports periodontitis as an important risk factor for CVD including stroke [70], peripheral artery disease [65, 71], and coronary heart disease [72]. Particularly, the inflammatory response accompanying periodontitis has been proposed as an important factor that may exert adverse effects on the regulation of BP (Table 2). The level of serum high-sensitivity CRP (hs-CRP), an acute-phase reactant that has been reported to predict the outcome of CVD, was found to be more increasing in patients with periodontitis than in control subjects, and it decreased significantly after periodontal treatment [73–75].

The association of CRP with hypertension in the setting of periodontitis has not been consistent, possibly due to many other factors that can elevate inflammatory markers, or simply hypertension itself is a multifactorial disease. However, it has recently been proposed that hs-CRP may be a useful marker linking periodontal disease and chronic inflammation [76] which leads to endothelial dysfunction [69]. Periodontitis has been reported to attenuate endothelium-dependent vasodilatation in experimental rats. This ill-effect was due to the elevation of systemic inflammatory biomarkers (CRP and IL-6), worsening the lipid profile, and increased production of vascular superoxide radicals and reduction of vascular nitric oxide synthase-3 (NOS-3) expression [69]. Furthermore, periodontitis is not confined to a localized lesion but may contribute to an increased systemic immune response in patients [77]. Periodontitis may therefore be capable to induce vascular inflammation which leads to endothelial dysfunction, an initial step for CVD. Al-Ghurabei [78] has documented that serum levels of hs-CRP and IL-6 were significantly elevated in patients with chronic periodontitis as compared to healthy control group. On the other hand, Vidal et al. [79] demonstrated that periodontal treatment reduces IL-6, CRP, and fibrinogen levels in patients having hypertension and severe periodontitis. Thus, it is getting clearer that inflammation might provide a potential link between hypertension and periodontitis.

### 4.2. Oral Infection

Periodontal bacterial infection may also be involved, at least in part, in the development of hypertension. Periodontitis results from the accumulation of bacterial species in subgingival biofilm, particularly by Gram-negative anaerobic and microaerophilic bacteria, such as *P. gingivalis, Prevotella intermedia, Prevotella nigrescens, Tannerella forsythia, Treponema denticola, Fusobacterium nucleatum, Aggregatibacter actinomycetemcomitans,* and *Campylobacter rectus *[33–35]. These periodontal pathogens are able to destruct and invade gingival tissues by proteolysis then enter the systemic circulation, causing transient bacteraemia [36]. Subsequently, the periodontal microbes may directly invade the arterial wall and lead to vascular inflammation and atherosclerosis [36].

Marcelino et al. [80] reported that *P. gingivalis* is the most prevalent bacterium harboured in atheromas, with its presence found in 50% of the atheroma samples obtained from patients with periodontitis. Infection of macrophages with *P. gingivalis* itself, and its outer membrane vesicles, is able to induce higher levels of foam cell formation [81]. *P. gingivalis* and its vesicles not only promote low-density lipoprotein (LDL) binding to macrophages but also induce macrophages to modify native LDL, which plays an important role in foam cell formation and the pathogenesis of atherosclerosis [81]. The wild-type strain has been shown to adhere to and enter human macrophages, suggesting the ability of *P. gingivalis* to invade macrophages may play an important role in its atherogenic potential [82].

Moreover, *P. gingivalis* has been demonstrated to induce the expression of cell adhesion molecules including ICAM-1, VCAM-1, P-selectin, and E-selectin [83]. *P. gingivalis* is reported to cause activation of endothelial cells and platelets which are the hallmark of atherogenesis [84]. Activation of endothelial cells is also involved in the pathogenesis of hypertension. Therefore, periodontopathogens from periodontal lesions into the circulation may deliver virulent factors to the arterial wall to initiate and/or promote foam cell formation in macrophages, thus contributing to development of CVD.

### 4.3. Oxidative Stress

Reactive oxygen species (ROS) such as superoxide anions and hydrogen peroxides are chemically reactive molecules. They damage cellular components including lipid membranes, nucleic acids, and proteins. ROS are formed as natural by-products during physiological processes in cell membranes, mitochondria, and endoplasmic reticulum. In addition, ROS can be generated from tobacco, pollutants, drugs, and ionizing radiation. However, excessive production of ROS leads to oxidative stress with an increase in the formation of free radicals as well as a decrease in antioxidant levels.

There is a growing evidence which indicates that periodontitis induces excessive production of ROS in periodontal tissue [85–90]. Therefore, oxidative stress is suggested to be involved in the pathogenesis of periodontal tissue destruction. Clinical studies have reported that periodontitis is correlated with increased lipid peroxidation in saliva and gingival crevicular fluid [85, 87, 89]. In addition, experimental studies revealed that oxidative damage on periodontal tissue was due to periodontitis [86] along with an elevation of hydrogen peroxide in polymorphonuclear leukocytes [90].

As the condition of periodontitis worsens, the production of ROS increases in response to periodontal inflammation; subsequently ROS enter the systemic circulation [91, 92]. With such, oxidation of biomolecules leads to circulating oxidative stress, which may damage various organs. Hence, the increase in circulating oxidative stress elicited by periodontitis may cause detrimental effects on systemic health.

Oxidative stress induced by locally infiltrating immune cells also participates in hypertension. Oxidative stress has been implicated in the development of hypertension. ROS are widely accepted as the mediators for vasoconstriction and vascular inflammation, and bioavailability of NO is strongly related to hypertension [93, 94]. Reactive oxygen radicals produced from the destruction of periodontal tissue cause an imbalance between oxidant and antioxidant activities. Furthermore, bacterial endotoxins such as lipopolysaccharides may promote oxidative stress mediated by mitochondrial dysfunction that lowers coenzyme Q_10_ level and citrate synthase activity, which further increase the rate of free radical production [95].

Imbalance between oxidant and antioxidant activities within the oral cavity negatively affects systemic oxidant status, as reflected by reduced antioxidant capacity [96]. D'Aiuto et al. [97] demonstrated that patients with periodontitis had higher diacron-reactive oxygen metabolites (D-ROM) and lower total antioxidant scavengers compared to healthy controls. The findings were independent of matching age and gender, as well as other confounders including ethnicity, smoking, and standard lipid profiles [97].

### 4.4. Endothelial Dysfunction

NO is released by the endothelium in order to regulate homeostasis of vascular system. High BP has been suggested to be associated with the imbalance between antioxidant and ROS production [98–100]. The impairment of endothelium-dependent relaxation is observed in hypertensive subjects and experimental models [101–106]. This occurrence may be due to a reduction in NO bioavailability, either via a decrease in production or an increased deactivation by ROS in the vascular wall. Peroxynitrite, a cytotoxic prooxidant resultant from the reaction between NO and ROS, is able to compromise endothelial integrity.

Periodontal disease may contribute to endothelial dysfunction [107], which eventually increases the risk of hypertension. NO deficiency is strongly related to the redox imbalance [108]. Inducible nitric oxide synthase (iNOS), which is expressed exclusively under inflammatory condition to produce large amounts of prooxidative NO, is prominently expressed in gingival tissues with periodontitis [109]. Furthermore, given that inflammation or even oxidative stress can destruct extracellular matrix (ECM) [110, 111], it is possible that periodontitis may link to adverse vascular remodelling. Damage to the ECM has been shown to cause structural and functional alterations, consequently affecting cell adhesion, proliferation, and signalling pathway. Hence, this impairment of elastic properties of large arteries plays a significant part in the development and progression of hypertension.

Various studies documented the involvement of endothelial dysfunction in periodontitis with the presence of inflammatory biomarkers. Seinost et al. [112] showed that flow-mediated dilation was lower in patients with periodontitis than in the control in addition to an elevation in CRP concentration. Higashi et al. [65] reported that periodontal therapy reduced serum concentrations of CRP and IL-6 and enhanced acetylcholine-induced vasodilatation in patients with periodontitis. Severe periodontitis is associated with significant endothelial dysfunction that is reversible after successful periodontal therapy in hypertensive patients [113]. Elter et al. [114] also demonstrated that periodontal treatment significantly improved endothelial function, serum CRP, and IL-6 levels.

## 5. Conclusion

In summary, the current epidemiological data, mainly from cross-sectional studies, show an association between hypertension and periodontitis. However, there is no strong proof to indicate that a causal relationship exists. In order to connect the relationships between dentistry and medicine, additional issue needs to be addressed for the improvement in managing the overall health of patients. Future studies should be conducted to yield better understanding of the mechanisms and interactions between hypertension and periodontitis, which will further strengthen the involvement between dental and medical communities. Since previous studies demonstrated an elevation in BP which is associated with periodontitis, preventive approaches targeted at reducing BP should also be included in the management of periodontitis. Periodontal health is achievable in both individual level as well as the population level. These preventive measures should be emphasized in oral health promotion programme, in order to enhance overall health outcomes.

## Figures and Tables

**Table 1:** Studies demonstrating significant association of hypertension and periodontitis.

Study	Year	Country studied	Sample size	Type of study	Findings	Adjusted for
Ogawa et al. [45]	1998	Japan	2000	Cross-sectional	Hypertensive subjects had higher CPITN	None

Angeli et al. [46]	2003	Italy	104	Cross-sectional	↑ SBP with ↑ periodontitis severity	None

Taguchi et al. [47]	2004	Japan	98	Cross-sectional	↑ SBP (*P* = 0.058), ↑ DBP (*P* = 0.021), hypertension in subjects with missing teeth (OR: 3.59; CI: 1.1–11.7)	Obesity, hypercholesterolemia, hypertriglyceridemia

Inoue et al. [48]	2005	Japan	364	Cross-sectional (2 time points)	Periodontitis associated with ↑ BP and WBC count	Age, gender, BMI, smoking, drinking, hypertension, DM, WBC count

Holmlund et al. [42]	2006	Sweden	4254	Cross-sectional (Retrospective)	Periodontal pocket related to hypertension (*P* < 0.0001), ↑ periodontitis severity with hypertension (OR: 1.32; CI: 1.13–1.54; *P* < 0.0005)	Age, gender, number of teeth, smoking

D'Aiuto et al. [49]	2006	England	40	Prospective intervention randomized controlled trial	↓ 7 ± 3 mmHg of SBP after 2 months of intensive treatment	None

Völzke et al. [50]	2006	Germany	4185	Cross-sectional (SHIP)	↑ SBP (*P* < 0.05) and OR: 1.91 (CI: 1.21–3.02; *P* < 0.05) for hypertension in male with 0–6 teeth compared to fully dentate	Age, BMI, education, smoking, diet, DM, antihypertensive medication

Engström et al. [43]	2007	Sweden	390	Cross-sectional	DBP associated with deep periodontal pockets	Age, gender, tobacco use, number of teeth

Völzke et al. [51]	2007	Germany	1913	Cross-sectional (SHIP)	↑ SBP (female: 11.7 mm Hg; male: 5.7 mmHg) in edentulous compared to fully dentate	None

O. A. Ayo-Yusuf and I. J. Ayo-Yusuf [52]	2008	South Africa	9098	Cross-sectional data (SADHS)	Higher SBP (12 mmHg) and DBP (5 mmHg) in complete edentulous compared to fully dentate OR: 1.35 (CI: 1.02–1.78) for hypertension in complete edentulous	Age, BMI, DM, education, income, diet, alcohol, smoking, family history of hypertension, oral hygiene behaviour (daily brushing, dental visits)

Franek et al. [53]	2009	Poland	99	Cross-sectional	Periodontitis severity associated with central BP and pulse pressure (*P* < 0.05)	Age, gender, BMI, hypertension duration, smoking, number of drugs taken

Fujita et al. [54]	2009	Japan	54551	Cross-sectional data	Female (OR: 1.52; CI: 1.14–2.03; *P* = 0.005); male (OR: 1.24; CI: 1.06–1.45; *P* = 0.006) for hypertension in no brushing compared to brushing after every meal	Age, BMI, smoking, alcohol, walking time

Nesse et al. [55]	2010	The Netherlands	1208	Cross-sectional	↑ hypertension prevalence in periodontitis subjects compared to controls (*P* = 0.001)	None

de Oliveira et al. [56]	2010	Scotland	11869	Cross-sectional (Scottish Health Survey)	↑ hypertension prevalence in subject with rare teeth brushing (*P* < 0.001)	None

Morita et al. [57]	2010	Japan	1023	Prospective cohort	Periodontal pocket associated with hypertension (OR: 1.5; CI: 1.0–2.3)	Age, gender, smoking, regular exercise, eating between meals, healthy body weight

Franek et al. [58]	2010	Poland	155	Cross-sectional	Periodontitis severity associated with central SBP (*P* = 0.011) & DBP (*P* = 0.006)	Age, gender, BMI, hypertension and insulin treatment

Tsakos et al. [59]	2010	United States of America	11948	Cross-sectional data (NHANES III)	↑ SBP (*P* < 0.001) OR: 1.1 (CI: 1-1.1; *P* < 0.05) for hypertension in ↑ 10% gingival bleeding	Age, gender, BMI, ethnicity, CRP, creatinine, Na^+^/K^+^ ratio, chronic conditions, smoking, alcohol, education, income

Desvarieux et al. [60]	2010	United States of America	653	Cross-sectional data (INVEST)	↑ SBP & DBP OR: 3.13 (CI: 1.62–6.03; *P* < 0.001) for hypertension when etiological bacterial burden is high	Age, gender, BMI, race, education, smoking, DM, LDL-C, HDL-C, nonetiological periodontal bacteria

Zhang et al. [61]	2011	Xinjang Uygur	1415	Cohort	Periodontitis associated with hypertension (OR: 1.75; CI: 1.30–2.36; *P* < 0.01)	Age, gender, BMI, waist circumference, glycometabolism disorder, hyperlipidemia, chronic kidney disease

Vidal et al. [62]	2011	Brazil	137	Case-control	Hypertension associated with severe chronic periodontitis (OR: 4.04; CI: 1.92–8.49), with generalized chronic periodontitis (OR: 2.18; CI: 1.04–4.56)	Gender, race, DM, alcohol, smoking

Peres et al. [63]	2012	Brazil	1720	Cross-sectional	Edentulous subjects had a SBP 8.3 mmHg (CI: 0.1–16.7) higher than those with 10 or more teeth in both dental arches	Age, gender, BMI, education, income, smoking, alcohol, DM, leisure physical activity, use of dental prosthesis, self-rated health status

Rivas-Tumanyan et al. [64]	2013	Puerto Rica	182	Cross-sectional	Periodontitis severity associated with high BP (OR: 2.93; CI: 1.25–6.84) OR: 4.20 (CI: 1.28–13.80) restricted to those with hypertension history and/or taking antihypertensive medications	Age, gender, smoking, drinking

Symbols indicate: ↑: increased; ↓: decreased.

BMI: body mass index; CI: confidence interval; CPITN: community periodontal index of treatment needs; CRP: C-reactive protein; DBP: diastolic blood pressure; DM: diabetes mellitus; HDL-C: high-density lipoprotein cholesterol; INVEST: Oral Infections and Vascular Disease Epidemiology Study; LDL-C; low-density lipoprotein cholesterol; NHANES: National Health and Nutrition Examination Survey; OR: odds ratio; SADHS: South African Demographic and Health Survey; SBP: systolic blood pressure; SHIP: Study of Health in Pomerania; WBC: white blood cell.

**Table 2:** Studies reporting inflammatory involvement in the interrelationship between hypertension and periodontitis.

Study	Design	Periodontal evaluation	Inflammatory markers	Hypertension-related parameters	Key findings
Higashi et al. 2009 [65]	Prospective	PPD CAL GB	CRP IL-6	Forearm blood flow responses to vasoactive substances, SBP and DBP	Periodontitis is associated with reduced NO bioavailability and ED, with systemic inflammation as a predictor of ED

Papapanagiotou et al. 2009 [66]	Case-control	Reported periodontitis	Leukocyte counts Platelet count CRP P-selectin	SBP and DBP	Periodontitis is associated with platelet activation

Herrera et al. 2011 [67]	Experimental	X-ray bone loss assessment	MPO TBARS NT	NO	NO contributes to the systemic effects of periodontitis

Eder et al. 2012 [68]	Case-control	Radiography (WHO)	PGE_2_ TXB_2_	PGI_2_	Presence of granuloma in periodontitis is linked to inflammation and the synthesis of metabolites of AA

Brito et al. 2013 [69]	Experimental	Measurement of alveolar bone loss of mandibles	Leukocyte counts IL-6 CRP	Mean arterial pressure, vascular reactivity	Periodontitis induces systemic and vascular inflammation which lead to ED

AA: arachidonic acid; CAL: clinical attachment loss; CRP: C-reactive protein; DBP: diastolic blood pressure; ED: endothelial dysfunction; GB: gingival bleeding; IL: interleukin; MPO: myeloperoxidase; NO: nitric oxide; NT: nitrotyrosine-containing protein; PGE_2_: prostaglandin E_2_; PGI_2_: prostacyclin; PPD: probing pocket depth; SBP: systolic blood pressure; TBARS: thiobarbituric acid reactive substances; TXB_2_: thromboxane B_2_; WHO: World Health Organisation.
